# Long-term outcomes of moderately hypofractionated radiotherapy (67.5 Gy in 25 fractions) for prostate cancer confined to the pelvis: a single center retrospective analysis

**DOI:** 10.1186/s13014-020-01679-0

**Published:** 2020-10-02

**Authors:** Lihong Yao, Jianzhong Shou, Shulian Wang, Yongwen Song, Hui Fang, Ningning Lu, Yuan Tang, Bo Chen, Shunan Qi, Yong Yang, Hao Jing, Jing Jin, Zihao Yu, Yexiong Li, Yueping Liu

**Affiliations:** 1 Department of Radiation Oncology, National Cancer Center/National Clinical Research Center for Cancer/Cancer Hospital, Chinese Academy of Medical Sciences (CAMS) and Peking Union Medical College (PUMC), Beijing, 100021 China; 2 Department of Gynecologic Oncology, Beijing Obstetrics and Gynecology Hospital, Capital Medical University, Beijing, 100006 China; 3 Department of Urology, National Cancer Center/National Clinical Research Center for Cancer/Cancer Hospital, Chinese Academy of Medical Sciences (CAMS) and Peking Union Medical College (PUMC), Beijing, 100021 China

**Keywords:** Prostate cancer, Moderate hypofractionation, Long-term outcomes, Treatment toxicities

## Abstract

**Background:**

There is an increasing application of moderately hypofractionated radiotherapy for prostate cancer. We presented our outcomes and treatment-related toxicities with moderately hypofractionated (67.5 Gy in 25 fractions) radiotherapy for a group of advanced prostate cancer patients from China.

**Methods:**

From November 2006 to December 2018, 246 consecutive patients with prostate cancer confined to the pelvis were treated with moderately hypofractionated radiotherapy (67.5 Gy in 25 fractions). 97.6% of the patients received a different duration of androgen deprivation therapy. Failure-free survival (FFS), prostate cancer-specific survival (PCSS), overall survival (OS), and cumulative grade ≥ 2 late toxicity were evaluated using the Kaplan–Meier actuarial method. Prognostic factors for FFS, PCSS, and OS were analyzed.

**Results:**

The median follow-up time was 74 months (range: 6–150 months). For all patients, the 5- and 10-year FFS rates were 80.0% (95% CI: 74.7–85.7%) and 63.5% (95% CI 55.4–72.8%). The failure rates for the intermediate, high-risk, locally advanced, and N1 groups were 6.1%, 13.0%, 18.4%, and 35.7%, respectively (*P* = 0.003). Overall, 5- and 10-year PCSS rates were 95.7% (95% CI 93.0–98.5%) and 88.2% (95% CI 82.8–93.8%). Prostate cancer-specific mortality rates for the high-risk, locally advanced, and N1 groups were 4.0%, 8.2%, and 23.8%, respectively (*P* < 0.001). Overall, 5- and 10-year actuarial OS rates were 92.4% (95% CI 88.8–96.1%) and 72.7% (95% CI 64.8–81.5%). High level prostate-specific antigen and positive N stage were significantly associated with worse FFS (*P* < 0.05). Advanced T stage and positive N stage emerged as worse predictors of PCSS (*P* < 0.05). Advanced age, T stage, and positive N stage were the only factors that were significantly associated with worse OS (*P* < 0.05). The 5-year cumulative incidence rate of grade ≥ 2 late GU and GI toxicity was 17.8% (95% CI 12.5–22.7%) and 23.4% (95% CI 17.7–28.7%), respectively.

**Conclusions:**

Moderately hypofractionated radiotherapy (67.5 Gy in 25 fractions) for this predominantly high-risk, locally advanced, or N1 in Chinese patients demonstrates encouraging long-term outcomes and acceptable toxicity. This fractionation schedule deserves further evaluation in similar populations.

## Background

With a rapidly aging population and changing lifestyles, the incidence of prostate cancer in China has increased from 4.0 per 100,000 to 20.0 per 100,000 between 1990 and 2017 [1]. The prostate-specific antigen (PSA)-based screening program effectively detected early prostate cancer, and these patients can be cured using modern external beam radiotherapy (EBRT) or radical prostatectomy [2, 3]. However, PSA screening is not performed in China, and patients have predominantly high-risk, locally advanced, or metastatic prostate cancer, and are more difficult to cure or incurable. Definitive EBRT is a treatment choice for localized and locally advanced prostate cancer cases.

In the last decade, by using of contemporary high precision treatment delivery techniques such as image-guided three-dimensional conformal radiotherapy, intensive-modulated radiotherapy (IMRT), and proton therapy, the irradiation dose of target volume has been increased, the local control rate, and disease-free survival rate have been improved, while the normal surrounding tissue sparing has been enhanced [4–6]. Evidence from multiple retrospective and prospective series of patients with localized prostate cancer confirmed the theoretical benefits of dose escalation. However, dose escalation with conventional fractionated (1.8–2.0 Gy/fraction) EBRT results in more hospital visits for patients, a resource burden on the treatment facilities, and a high cost for society. On the basis of the α/β model for prostate cancer, a hypofractionated course of EBRT with larger fraction sizes and fewer treatments would potentially increase therapeutic benefits without increasing toxicity in the bladder and rectum [7, 8]. There is an increasing application of moderately hypofractionated radiotherapy for prostate cancer [9–13]. Studies of moderately hypofractionated radiotherapy for relatively early stage patients have demonstrated promising results which are comparable to conventionally fractionated regimens [14–16].

However, there are less data on long-term outcomes for patients treated with hypofractionated radiotherapy in China, especially for a relatively higher risk Chinese cohort. We previously reported preliminary results using moderately hypofractionated IMRT (67.5 Gy delivered at 2.7 Gy/fraction) for pelvic-confined prostate cancer from our center [17]. This time we presented the long-term outcomes of a relatively large number of patients treated with the same dose scheme from our center.

## Methods

### Patients

From November 2006 to December 2018, 246 consecutive patients with prostate cancer confined to the pelvis were treated with moderately hypofractionated radiotherapy (67.5 Gy in 25 fractions) at our institution. Patients with biopsy-proven adenocarcinoma of the prostate, stage T1-4N0-1M0, according to the American Joint Committee on Cancer staging system, with any Gleason score (GS), any PSA level, and with a World Health Organization performance status of 0–1 were selected for this study. Pre-treatment evaluation consisted of a complete medical history, digital rectal examination, bone scan, chest radiograph, computed tomography (CT) of the abdomen and pelvis, magnetic resonance imaging (MRI) of the prostate and blood work including serum PSA and testosterone, and liver and renal function tests. Risk stratification was performed using modified National Comprehensive Cancer Network (NCCN) 2017 criteria.

### Radiotherapy

All patients were instructed to empty the rectum, fill the bladder, and drink 1000 ml of water 1 h before the CT simulation and each treatment fraction. Patients were immobilized in the supine position using thermoplastic pelvic fixation. CT scans were performed from the superior border of the fourth vertebra to 5 cm inferior to the ischial tuberosities, with 3/5 mm slice thickness and spacing. To facilitate clinical target volume (CTV) and critical normal structures delineation, intravenous and oral contrast were administered. The CT images were transferred to a Pinnacle planning system (Philips, Netherlands) for contouring and treatment planning.

The CTV comprised the prostate alone for low-risk patients and the prostate and proximal 1.5–2.0 cm seminal vesicles for intermediate-risk patients. In high-risk and locally-advanced (T3b-T4) patients, CTV1 included the prostate and proximal 2.0–2.5 cm seminal vesicles, and CTV2 included the pelvic lymph nodes if its involvement probability ≥ 30% (estimated from Roach’s equation: LN% = (2/3) PSA + (GS − 6) × 10) [18]. For N1 patients, if residual pelvic lymph nodes (≥ 1 cm) were detected on CT or MRI after 4–6 months of neoadjuvant androgen deprivation therapy (ADT), a local concomitant boost was administered. The planning target volume (PTV/PTV1) was created by expanding the CTV/CTV1 0.5 cm posteriorly (prostate-rectal interface) and 0.7–1.0 cm in other directions. PTV2 encompassed the CTV2 with a margin of 0.7 cm.

The prescribed dose was 67.5 Gy in 25 fractions for PTV/PTV1, which was equivalent to a total dose of 81 Gy in 2 Gy/fraction, using an α/β ratio of 1.5 [19, 20]. The prescribed dose to the PTV2 was 45–50 Gy in 1.8–2.0 Gy/fraction. The local concomitant boost dose for residual metastatic pelvic lymph nodes was 60–67.5 Gy. The delineation for normal tissue structures and the dose constraints used for the target and normal tissues have been described previously [17]. IMRT (n = 145, 58.9%) using a 5- to 7-field coplanar beam arrangement, volumetric modulated arc therapy (VMAT, n = 90, 36.6%), or helical tomotherapy (HT, n = 11, 4.5%) was planned with an inverse planning technique. All patients were treated daily, 5 days a week. Image guidance of cone beam CT (CBTC) was performed daily during the first week, and twice a week thereafter in 78.9% (n = 194) of the patients. An electronic portal imaging device (EPID) was used on the remaining patients.

### Androgen deprivation therapy

ADT was administered at the discretion of the treating physician and consisted of surgical castration (n = 12) or luteinizing hormone-releasing hormone agonists combined with antiandrogens. Low-risk patients received no hormone therapy; intermediate-risk patients received 2–3 months of neoadjuvant hormone therapy, followed by concurrent then adjuvant therapy totaling 4–6 months (including 1 surgical castration patient); high-risk, locally advanced, and N1 patients were given 4–6 months hormone therapy neoadjuvantly and continued for up to 24–36 months. The median duration of androgen suppression for the intermediate-risk group was 10 months (range: 4–142 months), and 36 months (range: 1–154 months) for high-risk, locally advanced, and N1 patients.

### Evaluation and follow-up

Patients were evaluated at regular intervals after treatment (every 3 months during the first 2 years, every 6 months in the third to fifth years, and annually thereafter). The follow-up evaluation included patient symptom assessment at each visit, physical examination, serum PSA measurements, and imaging studies when necessary. During each patient interview, gastrointestinal (GI) and genitourinary (GU) toxicity were assessed and graded according to Common Terminology Criteria for Adverse Events, version 4. Late toxicity was defined as any toxicity documented 3 months after the end of radiotherapy. The cumulative worst-grade toxicity was documented for each patient.

### Statistical analysis

All patients who were unable to follow-up were censored at their last follow-up visit. Biochemical failure was defined according to the Radiation Therapy Oncology Group and American Society for Radiation Oncology’s Phoenix definition as nadir plus 2.0 ng/ml [21]. Clinical failure was defined as local and/or distant progression detected by any image-based examinations. Failure-free survival (FFS) including PSA relapse-free survival, clinical failure-free survival, or both, was calculated from the date of radiation completion to the date of last follow-up without any failure.

FFS, prostate cancer-specific survival (PCSS), overall survival (OS), and incidence probabilities of cumulative grade ≥ 2 late toxicity were evaluated by the Kaplan–Meier actuarial method. Univariate and multivariate Cox-regression analyses were done to assess the prognostic factors for FFS, PCSS and OS, including age at diagnosis, pre-ADT PSA level, biopsy GS, clinical T-stage, and clinical N-stage. In the univariate analysis, the difference between groups was compared by using the log-rank test. The variables with *P* < 0.1 in univariate analysis were included in multivariate analysis. A *P* value of < 0.05 was considered statistically significant. In all statistical analysis, the *P* values were two-sided. Statistical analyses were performed using R version 3.6.1.

## Results

### Patient and treatment characteristics

Baseline characteristics of patient and treatment are shown in Table 1. The median patient age was 71 years (range: 51–87 years) and the median pretreatment PSA was 31.8 ng/ml (range: 1.14–2376 ng/ml), with 158 patients (64.2%) having a level ≥ 20 ng/ml. 95 patients (38.5%) had GS 8–10 prostate cancer, and 43.1% (n = 106) of the patients had clinical T3–4 stage diseases, and 17.1% (n = 42) had pelvic lymph node-positive disease at initial diagnosis. All the N1 patients received pelvic lymph node irradiation, and 2, 54, and 38 patients from the intermediate-, high-risk, and locally advanced groups, respectively, also received prophylactic pelvic lymph node irradiation. Pelvic lymph node boost irradiation was administered for 33 N1 patients.

**Table 1:** Baseline characteristics

Characteristic	Number of patients (%)
Age (years)	
Median (range)	71 (51–87)
Gleason score	
≤ 6	55 (22.4)
7	96 (39.0)
8	51 (20.7)
9	37 (15.0)
10	7 (2.8)
PSA before treatment (ng/ml)	
Median (range)	31.8 (1.14–2376)
≤ 10	35 (14.2)
10–20	53 (21.5)
> 20	158 (64.2)
Clinical T stage	
T1	15 (6.1)
T2	125 (50.8)
T3	82 (33.3)
T4	24 (9.8)
NCCN risk group	
Low risk	6 (2.4)
Intermediate risk	49 (19.9)
High risk	100 (40.7)
Locally advanced^a^	49 (19.9)
N1^b^	42 (17.1)
ADT duration (months)	
Median (range)	31 (1–154)
≤ 6 months	22 (8.9)
> 6 and ≤ 12 months	30 (12.2)
> 12 and ≤ 24 months	49 (19.7)
> 24 and ≤ 36 months	81 (32.9)
> 36 months	58 (23.6)
Irradiation field	
Prostate + seminal vesicles	110 (44.7)
Prostate + seminal vesicles + pelvic lymph node	103 (41.9)
Prostate + seminal vesicles + pelvic lymph node + residual pelvic lymph node boost	33 (13.4)
Pelvic lymph node boost dose	
60 Gy/2.4 Gy/25f	10 (4.0)
62.5 Gy/2.5 Gy/25f	2 (0.8)
65 Gy/2.6 Gy/25f	2 (0.8)
67.5 Gy/2.7 Gy/25f	19 (7.7)

*ADT* androgen deprivation therapy, *PSA* prostate-specific antigen, *NCCN* National Comprehensive Cancer Network, *EBRT* external beam radiation therapy

^a^Locally advanced = T3b–T4

^b^N1 is defined as lymph nodes within pelvis with minimum diameter ≥ 1 cm detected on CT or MRI at initial diagnosis

### Survival outcomes

**Fig. 1:**
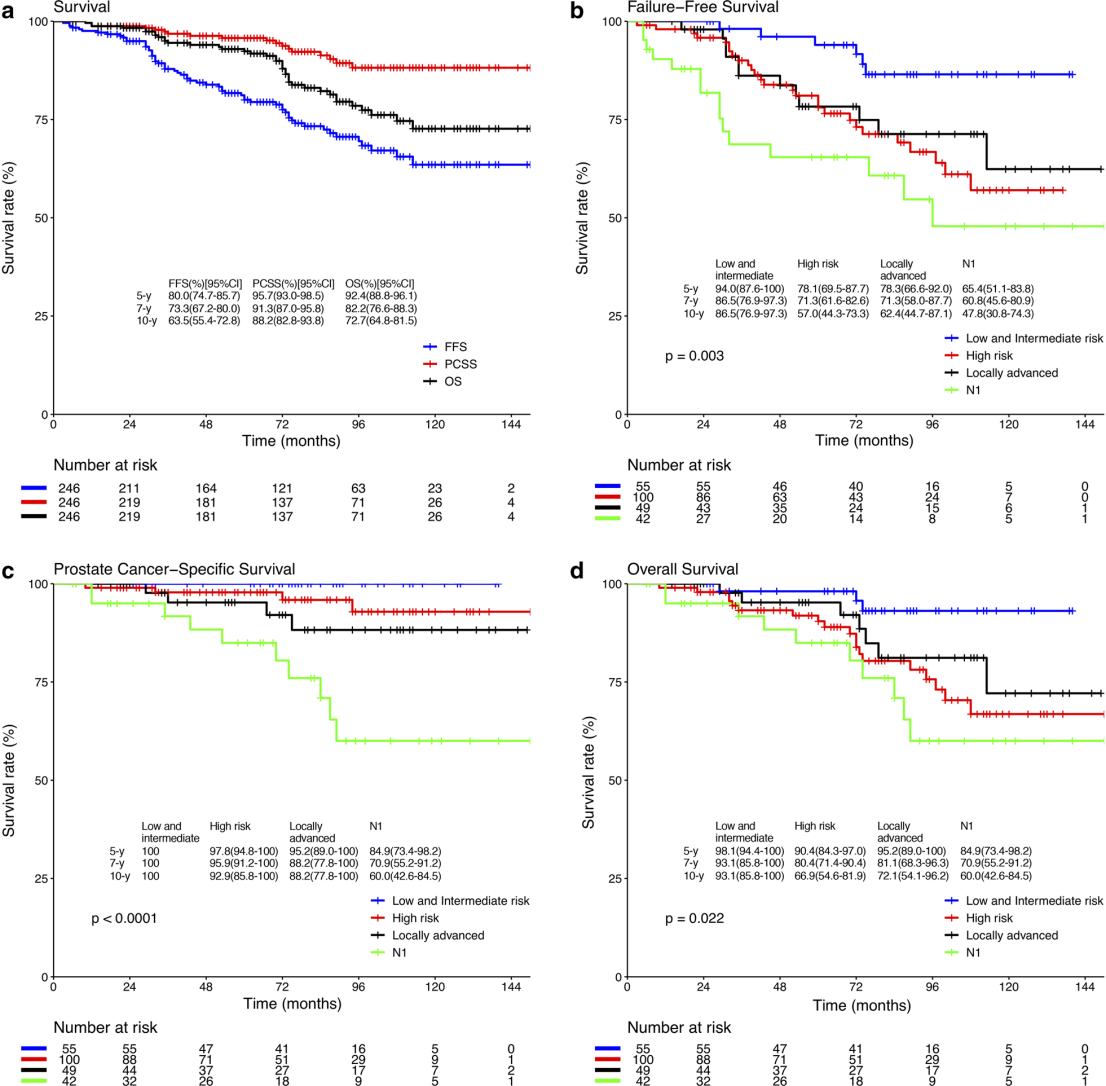
Kaplan–Meier curves. **a** The overall FFS, PCSS, and OS. **b** FFS by risk group. **c** PCSS by risk group. **d** OS by risk group

The median follow-up time was 74 months (range: 6–150 months). 19 patients (7.7%) had biochemical failures alone, 21 patients (8.5%) had biochemically detected and clinically radiographic evidence of distant failure, and 13 patients had bone metastases. No patient developed a clinical in-field relapse. The median time to failure was 33.5 months (range: 3–96 months). For the entire cohort, the 5- and 10-year FFS rates were 80.0% (95% CI 74.7–85.7%) and 63.5% (95% CI 55.4–72.8%) (Fig. 1a). No patient with low-risk disease experienced biochemical or clinical failure. Failure rates were 6.1%, 13.0%, 18.4%, and 35.7% for the intermediate-, high-risk, locally advanced, and N1 groups, respectively (*P* = 0.003) (Fig. 1b).

At the time of analysis, 39 patients (15.9%) had died, and 18 patients (7.3%) had died from prostate cancer. The median time to death was 70 months (range: 10–113 months). The 5- and 10-year PCSS rates were 95.7% (95% CI 93.0–98.5%) and 88.2% (95% CI 82.8–93.8%) (Fig. 1a), and the 5- and 10-year actuarial OS rates were 92.4% (95% CI 88.8–96.1%) and 72.7% (95% CI 64.8–81.5%) (Fig. 1a). No patient with low- and intermediate-risk disease died from prostate cancer. Prostate cancer-specific mortality rates were 4.0%, 8.2%, and 23.8% for the high-risk, locally advanced, and N1 groups, respectively (*P* < 0.001) (Fig. 1c). Eventually, 21 patients (8.5%) died of other causes during the follow-up period, and second primary cancer was the most common (n = 8) and the leading cause of death in our group. All the second primary cancers were outside of the irradiation field. The high-risk group had the highest number of deaths from other causes (n = 15). All the deaths from N1 patients were caused by prostate cancer. There were 1, 2, and 3 patients, respectively, who died of other causes in low-, intermediate-risk, and locally advanced group. Figure 1d presents the Kaplan–Meier curves for OS stratified by risk group.

In univariate analysis, early T-stage, negative N-stage, and decreasing baseline PSA were significant predictors of prolonged FFS (*P* = 0.034, 0.005, 0.039, respectively). Advanced T-stage, positive N-stage, and higher biopsy GS were associated with lower PCSS in univariate analysis (*P* < 0.001, < 0.001, = 0.012, respectively). Older age and positive N-stage were associated with lower OS in univariate analysis (*P* = 0.023, 0.042).

In multivariable analyses, high PSA level (> 20 ng/ml vs ≤ 10 ng/ml) and positive N-stage were significantly associated with worse FFS. Advanced T-stage (T4 vs T1–2) and positive N-stage emerged as significant predictors of worse PCSS. Finally, advanced age, T-stage (T4 vs T1–2), and positive N-stage were the only factors that were significantly associated with worse OS (Table 2).

**Table 2:** Multivariate Cox-regression analysis for FFS, PCSS and OS of the patients

Covariate	*β* value	*P* value	*HR*	95% CI for *HR*
FFS				
Clinical N stage				
N1/N0	0.672	0.028	1.958	1.074–3.568
PSA before treatment (ng/ml)				
10–20/ ≤ 10	0.925	0.152	2.521	0.711–8.938
> 20/ ≤ 10	1.195	0.047	3.305	1.018–10.729
PCSS				
Clinical T stage				
T3/T1–2	0.099	0.868	1.104	0.344–3.550
T4/T1–2	1.643	0.007	5.172	1.564–17.103
Clinical N stage				
N1/N0	1.778	< 0.001	5.917	2.219–15.780
Gleason score				
7/≤ 6	-0.229	0.800	0.795	0.135–4.677
≥ 8/≤ 6	0.764	0.354	2.147	0.427–10.790
OS				
Age				
≤ 70/ > 70	1.529	< 0.001	4.612	2.030–10.479
Clinical T stage				
T3/T1–2	0.325	0.360	1.384	0.690–2.773
T4/T1–2	1.591	0.003	4.908	1.708–14.106
Clinical N stage				
N1/N0	1.197	0.004	3.309	1.455–7.527
Gleason score				
7/≤ 6	0.090	0.854	1.095	0.420–2.855
≥ 8/≤ 6	0.387	0.411	1.472	0.581–3.729

*FFS* failure-free survival, *PCSS* prostate cancer-specific survival, *OS* overall survival, *β* regression coefficient, *HR* exponentiation of the *β* coefficient, other abbreviations as in Table 1

### Toxicity

**Fig. 2:**
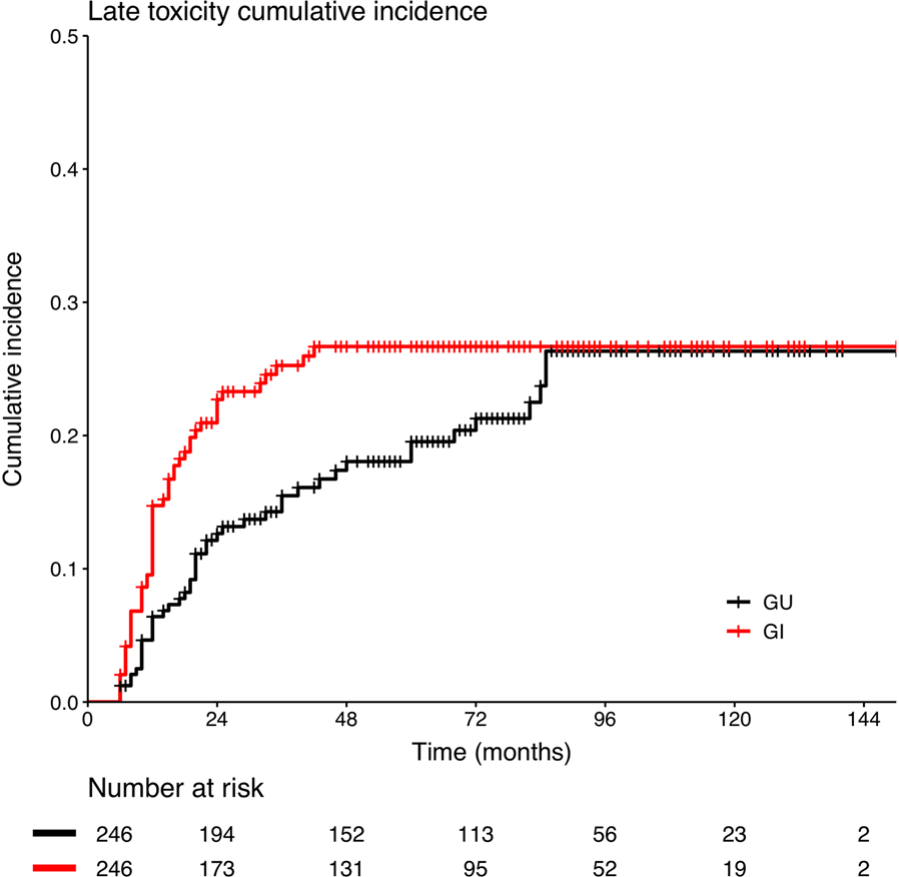
Cumulative incidence of grade ≥ 2 late genitourinary and gastrointestinal toxicity for the entire cohort

Toxicity data were available for all patients. Late GU and GI toxicities are summarized in Table 3. Grade ≥ 2 late GU toxicity was noted in 45 patients, commonly frequent of urination, incontinence, and dysuria, with a 5-year cumulative incidence rate of 17.8% (95% CI 12.5–22.7%) (Fig. 2). Of those, 12 patients experienced grade ≥ 3 late GU toxicity: 3 patients developed grade 3 toxicity of bleeding requiring hospitalization, 1 developed persistent grade 3 urinary incontinence, 3 developed grade 3 urinary frequency and nocturia, 4 developed grade 3 dysuria requiring cystostomy, and 1 developed grade 4 refractory hemorrhagic cystitis requiring cystostomy and blood transfusion. Grade ≥ 2 late GU toxicity cumulative incidence rates were not significantly different between patients with and without pelvic lymph node irradiation (*P* = 0.51).

**Table 3:** Late genitourinary and gastrointestinal toxicity (No.[%])

Grade	Late toxicity		GU toxicity		GI toxicity	
	GU	GI	Pelvis RT	No pelvis RT	Pelvis RT	No pelvis RT
0	148 (60.2)	138 (56.1)	79 (58.1)	69 (62.7)	74 (54.4)	64 (58.2)
1	53 (21.5)	54 (22.0)	32 (23.5)	21 (19.1)	27 (20.0)	27 (24.5)
2	33 (13.4)	48 (19.5)	19 (14.0)	14 (12.7)	30 (22.1)	18 (16.4)
3	11 (4.5)	5 (2.0)	5 (3.7)	6 (5.5)	4 (2.9)	1 (0.9)
4	1 (0.4)	1 (0.4)	1 (0.7)	0 (0)	1 (0.7)	0 (0)

*GU* genitourinary, *GI* gastrointestinal, *RT* radiation therapy

With respect to GI toxicity, 54 patients experienced grade ≥ 2 late GI toxicity, commonly hematochezia, frequent defecation, and fecal incontinence, with a 5-year cumulative incidence rate of 23.4% (95% CI 17.7–28.7%) (Fig. 2). Of those, grade ≥ 3 late GI toxicity was noted in 6 patients: 4 patients had grade 3 rectal bleeding requiring blood transfusion, 1 had grade 3 intestinal obstruction, and 1 patient had grade 4 rectal toxicity with rectovesical fistula formation requiring surgical intervention. A significant increase in cumulative grade ≥ 2 late GI toxicity occurred in the pelvic lymph node irradiation group (25.7% vs 17.3%, *P* = 0.0056).

## Discussion

Previously published hypofractionation radiotherapy studies, with different fraction and total doses, different toxicity scores, and different lengths of follow-ups have reported comparable outcomes and endurable toxicities, making it an attractive alternative to standard fractionation prostate radiotherapy. These studies have changed the clinical practice in many American and European medical centers. We have presented the long-term outcomes of a relatively large single-institution cohort of predominantly high-risk, locally advanced, and N1 Chinese prostate cancer patients treated with moderately hypofractionated radiotherapy and ADT. Our results showed satisfactory survival, good disease control, and well-tolerated treatment-related toxicity. The characteristics of the current study are as follows: (1) Chinese prostate cancer patients with predominantly high-risk, locally advanced, and N1 diseases; (2) similar treatment strategy; (3) mature follow-up.

Given the excellent therapeutic outcomes, both in terms of disease control and incidence rates of toxicity, the adoption of alternative modern, dose-escalated fractionation regimens necessitates careful clinical validation prior to widespread implementation. This is particularly important for those with high-grade disease as it has been hypothesized that the α/β ratio for such disease may be higher than that for low-grade prostate cancer [22]. However, multiple fractionated schedules have been clinically implemented, with fraction doses ranging between approximately 2.4–10 Gy [23]. The applicability of the linear quadratic model to accurately calculate the biological equivalent dose in the setting of fraction sizes over 5 Gy remains uncertain [24, 25], which result in difficulty in direct comparison with more moderately hypofractionated regimens. Nevertheless, our results provide valuable information for other moderate hypofractionation regimens employing fraction doses of approximately 2.4–4 Gy, especially for patients with more advanced disease.

There are several phase III randomized trials comparing standard versus moderate hypofractionated radiation therapy using escalated doses. Some researchers recommended hypofractionated radiotherapy as a new standard of care for localized prostate cancer, while others could not confirm that hypofractionation was non-inferior for cumulative late toxicity compared with standard fractionation [14–16, 26, 27]. Lee et al. [15] randomized 1092 patients with low-risk prostate cancer to hypofractionated radiotherapy (70 Gy in 28 fractions) versus conventional radiotherapy (73.8 Gy in 41 fractions). After a median follow-up of 5.8 years, the estimated 5-year disease-free survival was 86.3% in the hypofractionated radiotherapy group and 85.3% in the conventional radiotherapy group. Late grade 2 and 3 GI and GU adverse events were increased (HR, 1.31–1.59) in hypofractionated radiotherapy patients. Although an increase in late GI and GU toxicity were observed in the hypofractionated radiotherapy cohort, they concluded that in men with low-risk prostate cancer, the efficacy of their hypofractionated schedule was not inferior to conventional radiotherapy.

The HYPRO trial enrolled 804 intermediate- or high-risk patients, and randomly assigned them to receive either standard fractionation with 39 fractions of 2 Gy (5 fractions per week, totally 78 Gy) or hypofractionation with 19 fractions of 3.4 Gy (3 fractions per week, totally 64.6 Gy) [26, 27]. 67% of the patients received concomitant ADT for a median duration of 32 months. After a median follow-up of 5 years, 5-year relapse-free survival was 80.5% for patients assigned hypofractionation and 77.1% for those allocated conventional fractionation. The incidence of grade ≥ 2 late GU toxicity at 3 years was 39% in the standard arm and 41.3% in the hypofractionation arm. In addition, cumulative grade ≥ 3 late GU toxicity was significantly higher in the hypofractionation group (19.0% vs 12.9%). As for the 3 year rate of late GI toxicity, this dataset was 17.7% for the standard group compared with 21.9% for the hypofractionation group. There was no significant difference between cumulative grade ≥ 3 late GI toxicity in the two groups (2.6% vs 3.3%). The researchers explained that no planning objectives or constraints for the bladder, hormonal therapy, median age of 71 years, high percentage of patients with baseline GU symptoms, and the use of patients’ self-assessment questionnaires, all contributed to the high incidence of late GU toxicity. The authors did not recommend their hypofractionated radiotherapy regimen as the new standard of care for patients with intermediate-risk or high-risk prostate cancer.

Our finding is more consistent with those reported from the same fraction dose of 2.7 Gy prostate radiotherapy combined with ADT. Pollack et al. [28] conducted a randomized trial to compare the efficacy of moderate hypofractionation radiotherapy (70.2 Gy in 26 fractions) with conventional radiotherapy (76 Gy in 38 fractions) in 303 favorable- to high-risk patients. With a median follow-up of 68.4 months, 5-year biochemical and/or clinical disease failure rate was 23.3% for hypofractionated IMRT and 21.4% for conventional fractionation IMRT (*P* = 0.745). The overall incidences of grade ≥ 2 late GU and GI reactions for hypofractionated IMRT were 44.9% and 18.1%, respectively. There were no statistically significant differences in late toxicity between the two arms. Recently, Abu-Gheida et al. [5] reported 10-year outcomes for 854 patients across all risk groups treated with daily image-guided IMRT delivered 70 Gy in 28 fractions at 2.5 Gy per fraction. 5- and 10-year biochemical relapse free survival for 244 high risk patients were 63% and 42%, 5- and 10-year clinical failure free survival were 87% and 72%, and 5- and 10-year prostate cancer-specific mortality were 9% and 15%. 5-year cumulative incidence rate for grade ≥ 3 late GU and GI toxicity for their whole patients were 1.3% and 1.2%.

In our study, 77.7% of the patients enrolled were high-risk or more advanced. Nevertheless, this fractionation schedule (67.5 Gy in 25 fractions) still achieved promising results, with the 5- and 10-year FFS of 80.0% and 63.5%, and the 5- and 10-year PCSS rates of 95.7% and 88.2%. Also, there were no clinical in-field recurrence cases in our group, and the clinical failure presented as distant metastasis, indicating that this dose segmentation scheme was reasonable for the control of local prostate lesions, although our total dose was lower than that reported by Pollack et al. [28]. In this study, none of our low- and intermediate-risk patients died of prostate cancer. However, 23.8% of N1 patients died of distant metastasis of prostate cancer. Therefore, for N1 patients, local treatment is not enough, but an effective approach of systemic treatment is indispensable.

Compared with the above reports, severe late GU and GI toxicity events in our study were also low, and eased over time in line with the results from other studies [5, 28, 29]. However, a slightly increased rate of late GI toxicity was observed in our study. A potential explanation might that we applied image guidance selectively, not daily throughout treatment. This was due to a heavy workload, tight medical resources, and high treatment costs. Furthermore, older age and the use of ADT might account for this higher trend [26, 30]. In addition, we selectively radiated the pelvic lymph nodes to 45–50 Gy for 55.3% patients and local concomitant boosts were administered for 33 N1 patients who had residual metastatic pelvic lymph nodes after neoadjuvant ADT. Based on our promising efficacy and acceptable toxicity, this dose-escalated moderately hypofractionation schedule appears to be beneficial to Chinese prostate cancer patients, and our data are likely generalizable to other countries where PSA screening is not routinely carried out and whose prostate cancer populations typically present with more advanced disease.

There are several limitations to our study. This is a single-institution, single-arm, retrospective study involving a Chinese population, ranging from low risk to N1 disease. As a result, target volume and ADT duration varied greatly. Secondly, N1 disease was detected using imaging (CT and/or MRI) and was not histologically confirmed. ^18^F-Choline-PET/CT was a promising diagnostic tool in the definition of clinical stage and decision-making strategy of treatment volumes when integrated with conventional staging imaging [31]. But ^18^F-Choline-PET/CT was not available in our hospital. Furthermore, toxicity outcomes were physician reported rather than patient reported. In addition, modern daily image-guided delivery techniques were selectively used in our patients. Finally, the median follow-up time was relatively short, and the sample size was relatively small for prostate cancer.

## Conclusions

Moderately hypofractionated radiotherapy (67.5 Gy in 25 fractions) for this predominantly high-risk, locally advanced, or N1 Chinese patients demonstrates encouraging long-term outcomes and an acceptable incidence of toxicity. This fractionation schedule deserves further evaluation in similar populations.

## Data Availability

The datasets used and analyzed during the current study are available from the corresponding author on reasonable request.

## Abbreviations

PSA: Prostate-specific antigen
EBRT: External beam radiotherapy
IMRT: Intensive-modulated radiotherapy
GS: Gleason score
CT: Computed tomography
MRI: Magnetic resonance imaging
NCCN: National Comprehensive Cancer Network
CTV: Clinical target volume
ADT: Androgen deprivation therapy
PTV: Planning target volume
VMAT: Volumetric modulated arc therapy
HT: Helical tomotherapy
CBCT: Cone beam CT
EPID: Electronic portal imaging device
GI: Gastrointestinal
GU: Genitourinary
FFS: Failure-free survival
PCSS: Prostate cancer-specific survival
OS: Overall survival
